# Interaction Between Ruminal Acetate Infusion and Diet Fermentability on Milk Fat Production in Dairy Cows

**DOI:** 10.3390/ani15131931

**Published:** 2025-06-30

**Authors:** Natalie L. Urrutia, Camila Muñoz, Emilio M. Ungerfeld, Claudia Cisterna, Kevin J. Harvatine

**Affiliations:** 1 Centro Regional de Investigación Remehue, Instituto de Investigaciones Agropecuarias, Osorno 5290000, Región de Los Lagos, Chile; 2 Centro Regional de Investigación Carillanca, Instituto de Investigaciones Agropecuarias, Temuco 4880000, Región de La Araucanía, Chile; 3 Departamento de Producción Animal, Facultad de Ciencias Veterinarias y Pecuarias, Universidad de Chile, La Pintana 8820808, Región Metropolitana, Chile; 4 Department of Animal Science, The Pennsylvania State University, University Park, PA 16802, USA

**Keywords:** acetate, lactation, dairy cow, milk fat synthesis

## Abstract

Acetate is naturally produced in the rumen through feed degradation and fermentation. It serves as a primary energy source for ruminants and as a key substrate for de novo fatty acid synthesis in the mammary gland. The interaction of exogenous acetate with different animal and dietary factors is an area of growing interest, as it may have significant implications for milk fat synthesis. This study aimed to assess the effect of two diet fermentability levels on the short-term response of lactation to acetate supplementation in dairy cows. Eight ruminally cannulated multiparous European Holstein cows were randomly assigned to treatments in a crossover design that tested the effect of diet fermentability, acetate supply, and their interaction. Using corn silage as the only forage source and a constant forage-to-concentrate ratio, high-fermentability (HF) and low-fermentability (LF) diets were formulated. Acetate supply was investigated by infusing ruminally 10 moles of sodium acetate/d (ACE) or an equimolar infusion of control (CON). Therefore, the treatments were as follows: LF + CON; LF + ACE; HF + CON; and HF + ACE. No interactions between acetate and diet fermentability were found on performance variables. Acetate infusion decreased dry matter intake (DMI), milk yield, and milk protein yield and content but did not affect milk fat yield; however, it increased milk fat concentration, and this response tended to be more pronounced in the HF diet. Acetate infusions increased plasma β-hydroxybutyrate in the HF diet, but not in the LF diet, and increased plasma non-esterified fatty acid, which was likely a lipolysis response to reduced DMI and decreased energy balance. This study demonstrates that acetate availability can be a constraint on mammary lipogenesis, even with adequate dietary fiber.

## Simple Summary

This study explores how additional acetate, a product of fiber digestion in cows, influences milk production when feeding diets with different fermentability levels. We found that acetate boosts milk fat content, especially in highly fermentable diets, and maintained milk fat yield. However, acetate reduced feed intake, milk yield, and protein content, which was unexpected. These findings highlight the importance of acetate in milk fat synthesis, although the mechanisms decreasing feed intake are unclear.

## 1. Introduction

Dairy cows obtain the majority of their energy requirements for maintenance and lactation from short-chain fatty acids (SCFAs) produced in the rumen by microbial fermentation of the diet [1]. Acetate is the most abundant SCFA produced in the rumen and provides up to 49% of the energy arising from SCFA metabolism [1,2]. Also, acetate is the main substrate for milk fat synthesis, as it provides the majority of the carbon and half the reducing equivalents needed for de novo lipogenesis [3]. Acetate supplied experimentally to dairy cows through ruminal infusions or mixed in the diet has shown variable effects on milk yield [4,5,6] but has consistently increased milk fat yield. Milk fat is important for many dairy products, and producers are paid based on milk fat in many milk markets across the world [7], providing an incentive to understand the impact of diets and the SCFA absorbed from the rumen on the regulation of milk fat synthesis.

We previously reported that short-term ruminal infusion of acetate linearly increased milk fat concentration and quadratically increased milk fat yield [8]. The response was observed on the first day of infusion, and milk fat yield was maximally increased at 10 moles of sodium acetate/d. Feeding 10 moles of sodium acetate per day in a total mixed ration (TMR) resulted in 90 and 200 g increases in milk fat yield, as well as increases of 0.12 to 0.43 percentage units in milk fat content, as compared to a control diet in the studies by Urrutia et al. [4], and Matamoros et al. [5], respectively. Given the variability in milk fat concentration and yield responses to acetate supply, it is of interest to identify and understand specific digestive and fermentation conditions under which increasing acetate supply would have the most significant impact.

A previous meta-analysis by Maxin et al. [6] reported a tendency (*p* = 0.08) for an interaction between acetate infusion and diet composition on milk fat response, with a greater response in milk fat concentration observed when feeding higher-concentrate diets. Increasing concentrate increases dietary starch and decreases fiber, enhancing diet fermentability [9]. Diets that are more fermentable result in greater propionate and less acetate produced in the rumen [10,11]. While acetate is the major precursor of milk fat, propionate provides carbons for the triglyceride backbone (glycerol), and through acting as the main gluconeogenic substrate in ruminants [12], it supplies reducing equivalents required for de novo lipogenesis when metabolized via the pentose phosphate pathway. Therefore, diet-induced changes in ruminal acetate and propionate produced from diet fermentation, as highlighted by Maxin et al. [6], potentially interact with exogenous acetate supplementation to enhance milk fat synthesis. Experimentally, higher-concentrate diets can be achieved either by adjusting the forage-to-concentrate ratio or by increasing the starch content of the concentrate portion of a TMR. Recently, Matamoros et al. [13] employed the first approach (forage-to-concentrate adjustment) by replacing corn silage and alfalfa haylage with ground corn and canola meal. However, they observed no interaction between exogenous acetate supply and dietary fiber level on milk fat content or yield. Instead, acetate supplementation independently increased milk fat content and yield by 8.6% and 10.5%, respectively, regardless of fiber content.

The approach of reducing the forage-to-concentrate ratio may decrease the intake of dietary fiber and therefore decrease chewing activity, saliva production, and ruminal pH [14,15]. These conditions are associated with milk fat depression, as a positive linear relationship between ruminal pH and milk fat has been reported [9]. Also, the growth of bacterial populations that synthesize milk fat-depressing biohydrogenation intermediates is favored at lower ruminal pH [16]. An alternative experimental approach, providing diets differing in fiber level without altering the forage-to-concentrate ratio, would allow us to investigate the specific effects of the interaction between exogenous acetate supply and diet fermentability with a lower background of rumen milk fat-depressing biohydrogenation intermediates and therefore lower milk fat synthesis inhibition.

Based on this rationale, our hypothesis was that acetate infusion would result in a greater increase in milk fat yield in a highly fermentable diet, compared to in a lower- fermentability diet, when the forage-to-concentrate ratio was maintained. This study provides experimental conditions with two diets differing in fermentability based on neutral detergent fiber (NDF) and starch content, but with the same forage-to-concentrate ratio, versus previous research that has evaluated the interaction of acetate with two levels of dietary fiber achieved through diets that differed in the forage-to-concentrate ratio [13].

The main objective of this study was to investigate the interaction between acetate supply and diet fermentability using diets with equal forage-to-concentrate ratios, in European Holstein Friesian dairy cows.

## 2. Materials and Methods

### 2.1. Experimental Design and Treatments

**Figure 1:**
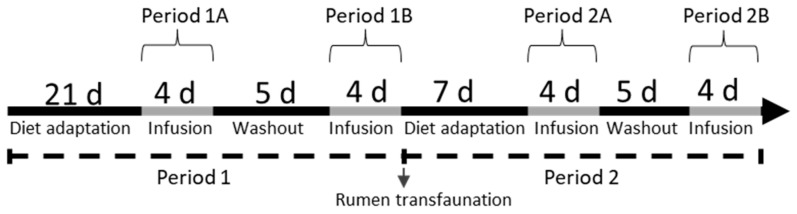
Schematic representation of experimental periods. In period 1, cows were fed either a high-fermentability (HF) or low-fermentability (LF) diet; in period 2, they were switched to the alternative diet. Following each adaptation phase, the cows received ruminal infusions of either 10 mol/d NaCl (CON) or sodium acetate (ACE), according to the infusion treatment sequence assigned.

Eight spring-calving, ruminally cannulated, multiparous European Holstein cows (120 ± 17.8 days in milk; 552 ± 56 kg body weight; mean ± SD) were randomly assigned to 1 of 2 dietary treatment sequences, and within each dietary period, they were assigned to 1 of 2 ruminal infusion treatment sequences in a crossover design that tested the effect of diet fermentability, sodium acetate infusion, and their interaction (n = 8). Using baled corn silage as the only forage source, two levels of diet fermentability were examined: high fermentability (HF, higher starch and lower NDF) and low fermentability (LF, lower starch and higher NDF). Acetate supply was investigated with short-term (4 d) ruminal infusion of 10 moles of sodium acetate/d (ACE) or an equimolar sodium chloride control (CON). Therefore, the treatments were as follows: (1) low-fermentability diet + control ruminal infusion (LF + CON); (2) low-fermentability diet + acetate ruminal infusion (LF + ACE); (3) high-fermentability diet + control ruminal infusion (HF + CON); (4) high-fermentability diet + acetate ruminal infusion (HF + ACE). Each dietary experimental period comprised 2 periods of 4 d infusions with 5 d of washout between ruminal infusions. A diet adaptation period of 21 d was originally planned for each dietary period (1 or 2) and was completed for the first dietary period (Figure 1). However, after the first dietary period was complete, ruminal transfaunation (90% of the rumen content, as in [17]) was performed between periods 1 and 2 to allow more rapid adaptation, and the second dietary adaptation period was reduced to 7 d. Switching rumen contents accelerates microbiota adaptation to diets [18,19]. Although shortening the second adaptation period was not originally planned, it was necessary because the corn silage available for this study was rapidly deteriorating (visual observation of mold and hot spots), and over 40% of it had to be discarded by the end of period 1. This arrangement resulted in 4 experimental periods as represented in Figure 1.

The TMR was formulated to meet the nutrient requirements of lactating dairy cows (100 days in milk, 550 kg of body weight, 25 kg/d of milk yield, and 3.5% of milk fat and 3.4% of milk protein) according to NRC [20], and to achieve 2 levels of diet fermentability based on their NDF and starch content (21.6 or 29.2% of starch and 38.4 or 32.4% of NDF, for the LF and HF diet treatments, respectively), without altering the forage-to-concentrate ratio. This was achieved by the substitution of corn grain for oat hulls based on the nutrient composition of ingredients sampled prior to the trial.

The dose of acetate was chosen based on the maximal milk fat yield response in a previous dose titration study [8]. The ACE ruminal infusate (2M) was prepared as described in Urrutia and Harvatine [21], by an initial dilution of 2288 mL of 100% anhydrous glacial acetic acid (Merck, Santiago, Chile) and 1536 g of NaOH pellets (Merck, Santiago, Chile) in 10 L of tap water, which was brought to a final volume of 20 L with tap water once the initial solution had cooled to room temperature. If necessary, the pH was adjusted to 6.2 with HCl or NaOH. The CON infusate was prepared by diluting 2340 g of NaCl in tap water to a final volume of 20 L, and the pH was adjusted to 6.2 with HCl. Control and ACE treatments provided equal sodium molarity (10 mol/d) and volume (5 L/d). Treatments were infused continuously at a rate of 3.47 mL/min, for 24 h/d over 4 d, through the rumen cannula using acid-resistant tubing (Norprene L/S 14, Masterflex, Cole-Parmer, Vernon Hills, IL, USA) and peristaltic pumps (L/S drive 7528-30 pump with Easy-Load 3 77800-60 pumphead, Masterflex, Cole-Parmer, Vernon Hills, IL, USA).

### 2.2. Animal Management and Diets

During diet adaptation and washout periods, cows were housed in groups by dietary treatment in free-stall accommodation. During ruminal infusion treatments, cows were housed in tie stalls. Cows were moved into tie stalls 2 d before the initiation of ruminal infusions to allow adaptation to the facility. Throughout the entire study, cows had ad libitum access to feed and water, and they were fed once daily (09:00 h) a TMR at 110% of the expected intake, and refusals were recorded daily.

Forage and TMR dry matter (DM) were determined weekly by drying in a forced-air oven at 55 °C for 72 h for diet adjustment and dry matter intake (DMI) determination. All diet ingredients were sampled weekly, dried, ground through a 1 mm sieve, and composited by period for nutrient analyses by wet chemistry procedures (Table 1). Briefly, the samples were analyzed for crude protein (CP; method 2001.11; [22]), NDF (amylase-treated NDF, method 2002.04; [22]), acid detergent fiber (ADF; method 973.18; [22]), starch (method 996.11; [22]), soluble carbohydrates (NFC; Method 14, [23]), ether extract (method 932.02; [22]), ash (method 923.03; [22]), Ca (method 985.35; [22]), P [24], and in vitro organic matter (OM) digestibility [25].

The resulting diets fed contained 21.2 and 16.3% starch and 34.6 and 38.6% NDF for the HF and LF diets, respectively (Table 1). A greater difference in dietary NDF and starch content was not possible to achieve due to the relatively high NDF content of the corn silage available for the study (46.3 to 49.8% NDF), likely because the silage was sourced from multiple bales (provided by the same supplier).

### 2.3. Milk Sampling and Analysis

Cows were milked twice daily at 07:30 and 17:30 h. During the adaptation periods, milk yield was determined by an integrated milk meter at the dairy parlor (DeLaval Alpro MM15; DeLaval International, Tumba, Sweden). When housed in the tie stalls, the cows were milked into a can with a mobile milking unit equipped with an integrated Waikato milk meter, and milk was directly weighed and recorded manually. Milk was sampled at both daily milkings starting from the day before until the end of the ruminal infusion periods, from the integrated milk meter, and preserved with bronopol and stored at 4 °C until milk fat and protein determination in a mid-infrared analyzer [26] (ISO 9622/IDF 141:2013, MilkoScan 4000, Foss Electric, Hillerød, Denmark). Also, a second subsample was collected at each milking from days 1 to 4 and stored at 4 °C. At the end of the infusion periods, the subsamples were pooled by cow and centrifuged at 1300× *g* for 20 min at 4 °C to obtain the upper fat layer or fat cake. The fat cake was stored at −20 °C, freeze-dried, and then subjected to fatty acid (FA) extraction with hexane and isopropanol, transmethylated with sodium methoxide, and quantified by GC with a flame ionization detector and a capillary column as in Rico and Harvatine [27], with slight modifications [SP-2560; 100 m × 0.25 mm (i.d.) with 0.2 μm film thickness; Supelco Inc., Bellefonte, PA, USA]. The initial oven temperature was 80 °C, and the oven temperature increased by 2 °C/min to 190 °C for 15 min and then increased by 5 °C/min to 215 °C for 3 min. The inlet and detector temperatures were 250 °C with a 50:1 split ratio. Peaks were identified using FAME standards (GLC 68D and 780 and pure trans-10,cis-12 CLA and cis-9,trans-11 CLA, Nu-Chek Prep Inc., Elysian, MN, USA; Bacterial Acid Methyl Ester Mix, 47080-U, Sigma-Aldrich Inc., St. Louis, MO, USA; and GLC 110 mixture, Matreya LLC., State College, PA, USA), and recoveries of individual FA were determined using an equal weight reference standard (GLC 461; Nu-Chek Prep Inc.).

### 2.4. Rumen and Blood Sampling and Analysis

On day 4 of infusions, blood samples were collected from a coccygeal vessel before feeding (09:00 h) and 6 h post feeding (15:00 h) in potassium EDTA and potassium oxalate/sodium fluoride vacuum tubes. Blood was immediately placed on ice, centrifuged within 45 min at 1300× *g* for 15 min at 4 °C, and plasma-harvested and stored at −20 °C until laboratory analysis. Plasma samples were analyzed for glucose [PGO Enzyme procedure no. P 7119; Sigma-Aldrich, St. Louis, MO, USA [28]], total non-esterified fatty acids [NEFA; Wako HR Series NEFA-HR kit, Wako Chemicals USA, Richmond, VA, USA as modified by Ballou et al. [29]], and β-hydroxybutyrate (BHB; using an AutoChemistry Analyzer LWC100 plus, Shenzhen Landwind Biomedical Technology Co., Ltd., Shenzhen, China).

Ruminal contents were collected at 09:30 h and 15:30 h on days 3 and 4 to represent before-feeding and near-maximal rumen fill (6 h after feeding). Samples were taken from 5 different locations in the rumen and composited. Solid and liquid fractions of ruminal samples were separated using a 4-layer gauze, and pH was recorded with a calibrated digital pH meter (model exStik^®^ PH100, Extech, Nashua, NH, USA).

### 2.5. Statistical Analysis

All data was analyzed using SAS software (Studio 3.81). Cow was the experimental unit, with n = 8 for all analyses. Performance responses, plasma metabolites, and rumen pH were analyzed as repeated measures in a mixed model. The full model tested included the fixed effects of diet, infusion treatment, time and all interactions, a covariate of initial performance in the case of performance responses, and the random effects of cow and period. For performance responses, time was day, and for plasma metabolites and rumen pH, time was AM or PM based on the significance (*p* < 0.05) of the terms in the model; a reduced model that excluded the diet × day and diet × day × infusion treatment interactions was used for performance variables. The subject was cow nested in period, and the Huynh–Feldt covariance structure was used. Denominator degrees of freedom were adjusted with the Kenwood–Rogers method.

Milk FA data was analyzed as a mixed model using ANOVA in SAS, including the fixed effects of diet, infusion treatment, and their interaction and the random effects of cow and period.

To evaluate the effects of the reduction in diet adaptation between dietary period 1 and 2, an initial screening of the fixed effect of period was included in all mixed models. The resulting *p*-values for all variables and models were non-significant.

For all analyses, least square means (LSMs) and average standard error (SE) are reported. Data points with Studentized residuals outside of ± 3.5 were considered outliers and excluded from analysis (resulting in 1 data point excluded from the BHB analysis). A protected LSD separation was used within each timepoint to compare treatment means when the treatment, or treatment by time interaction, was significant. Differences were declared significant at *p* ≤ 0.05 for main effects and at *p* ≤ 0.1 for interactions. Tendencies are reported for main effects at *p* ≤ 0.1 and for interactions at *p* ≤ 0.15.

## 3. Results

### 3.1. Dry Matter Intake, Milk Yield, and Composition

There were no interactions between diet fermentability and acetate infusion on DMI, milk yield, milk fat yield, milk protein content and yield, and apparent net energy balance (Table 2). The main effects of infusion treatment were detected for milk fat concentration and apparent net energy balance as follows: Milk fat concentration was increased by ACE (*p* < 0.001; Table 2), with an overall increase of 0.54 and 0.26 percentage units in the HF and LF diets and a tendency for an interaction between diet fermentability and acetate infusion (*p* = 0.12). Milk fat concentration tended to be greater by 0.15 percent units in the HF diet than in the LF diet (*p* = 0.08). Apparent net energy balance was reduced by ACE by 3.4 and 4.1 Mcal/d in the HF and LF diets, respectively (*p* = 0.03; Table 2). Milk fat yield was unaffected by treatments (*p* ≥ 0.66) due to the decrease in milk yield with ACE.

**Figure 2:**
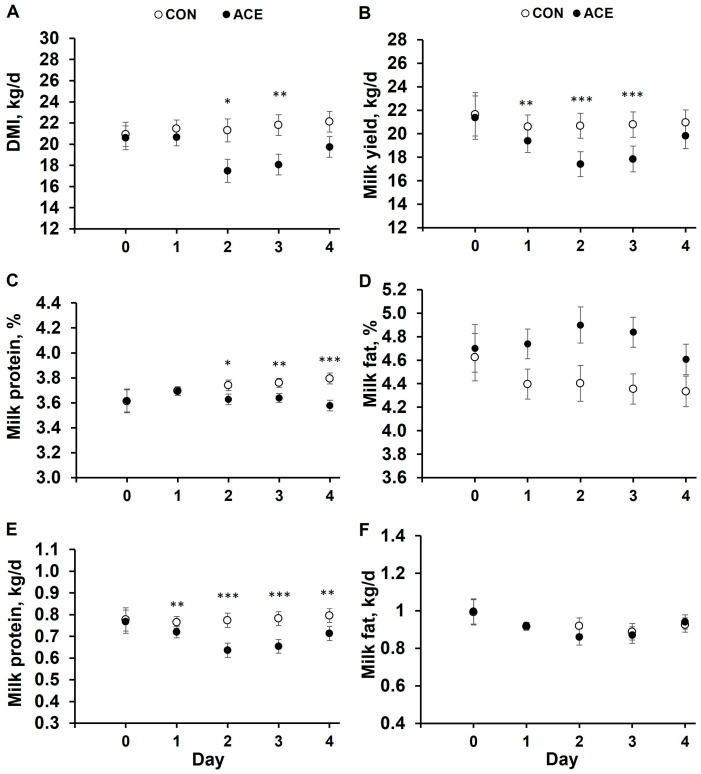
Time course of dry matter intake (Panel (**A**); kg DMI/d), milk yield (Panel (**B**); kg/d), milk protein content (Panel (**C**); %), milk fat content (Panel (**D**); %), milk protein yield (Panel (**E**); kg/d), and milk fat yield (Panel (**F**); kg/d) of cows ruminally infused with sodium acetate infusions. Treatments were 4 d continuous ruminal infusions of 10 moles/d of NaCl (CON) or 10 moles/d of sodium acetate (ACE) (n = 8 per treatment). Data shown corresponds to the LSM of the interaction between time (day) and ruminal infusion treatments. Symbols denote a significant difference in treatments within a timepoint (*: *p* ≤ 0.05; **: *p* ≤ 0.01; ***: *p* ≤ 0.001).

Interactions between infusion treatment and time were detected and will be described for milk yield, DMI, and milk protein content and yield (Figure 2). Milk yield was reduced by 6, 16, and 14% by ACE on days 1, 2, and 3 of the rumen infusions, compared to CON, and followed a similar pattern to the decrease in DMI (Figure 2). Milk protein concentration was reduced by ACE on days 2, 3, and 4 by 0.11, 0.12, and 0.22 percentage units, compared to CON (Figure 2), representing an overall 3% reduction. In our study, milk protein yield was reduced in ACE by 6, 18, 16, and 10%, on days 1, 2, 3, and 4, respectively, compared to CON (Figure 2).

### 3.2. Milk Fatty Acid (FA) Profile

No interactions between diet fermentability and acetate infusion on milk FA profile were observed; therefore, the main effects of treatments are presented (Table 3, Tables S1 and S2). Acetate infusion decreased the content of de novo-synthesized FA (g/100 g total milk FA) by 1.6 and 2.3 percentage units in the HF and LF diets, respectively (Table 3, *p* = 0.01). Conversely, milk FA of mixed origin increased in ACE as compared to CON by 3.9 and 1.5 percentage units in the HF and LF diets, respectively. This increase was primarily due to a higher concentration of C16:0, which rose by 3.9 and 1.5 percentage units in the HF and LF diets, respectively.

Preformed FA content was greater with the LF diet, with overall increases of 2.1 and 2.7 percentage units observed in the CON and ACE treatments, respectively (*p* = 0.04). Diet fermentability and ruminal acetate infusion affected milk stearic acid (C18:0) and total trans C18:1 content. An overall increase of 0.8 percentage units in C18:0 was observed in LF as compared to HF (*p* = 0.02), as well as a 0.81 percentage unit increase in ACE when compared to CON (*p* = 0.02). The total trans C18:1 content was 0.1 percentage units higher in the LF diet compared to the HF diet (*p* = 0.04) and 0.11 percentage units higher with ACE compared to CON (*p* = 0.03).

The yield and content of milk trans-11 C18:1 and the content of total trans C18:1 were increased by ACE. In the ACE treatment, the milk trans-11 C18:1 content overall increased by 0.1 percentage units as compared to CON (*p* < 0.001, Table 3), and the yield increased by 1 and 0.52 g per day in the HF and LF diets, respectively (Table S1). Also, the milk trans-11 C18:1 content was greater by 0.08 percentage units in the LF as compared to the HF diet (*p* = 0.002). Ruminal infusion of ACE reduced the content of odd- and branched-chain FA (OBCFA) by 0.42 and 0.16 percentage units in the HF and LF diets, respectively (*p* = 0.05). While diet fermentability and ruminal infusions influenced FA composition, neither factor affected the yield of FA from different sources (de novo-synthesized, mixed-origin, preformed) or the yield of OBCFA (Table 3).

### 3.3. Plasma Metabolites and Rumen pH

The plasma metabolites measured in the current study represent timeframes of metabolic states before (fasting state; AM) and 6 h after feeding (maximal fermentation products; PM). Plasma glucose was unaffected by diet or infusion treatments, and there was no interaction between these variables; however, there was a time effect, with plasma glucose being higher before feeding (AM) than at 6 h post feeding (PM; *p* = 0.001; Table 4).

**Figure 3:**
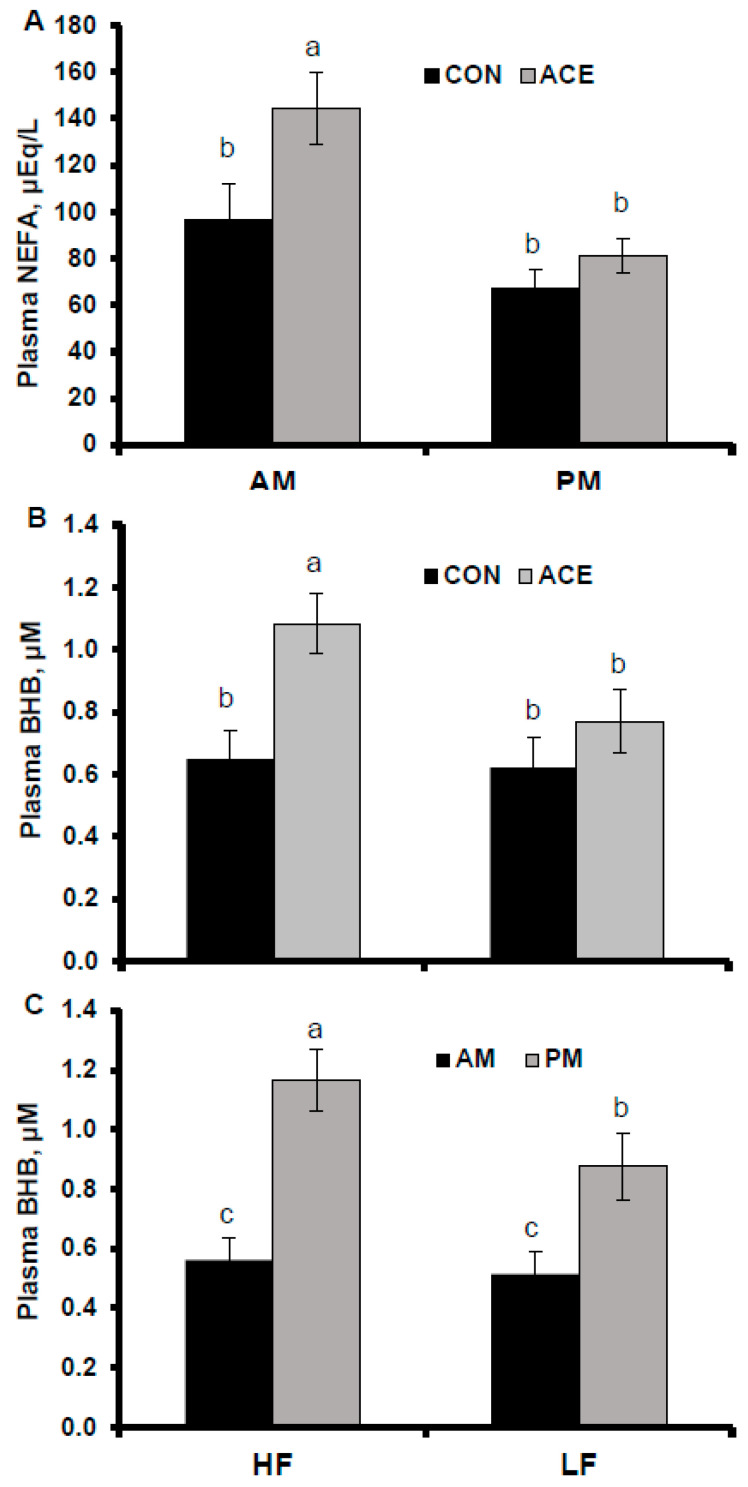
Interactions of ruminal infusions with time on plasma NEFA (**A**), diet fermentability with ruminal infusions (**B**), and time (**C**) on plasma BHB in lactating dairy cows. Treatments included diet fermentability (high fermentability (HF) or low fermentability (LF)); and ruminal infusions of 10 moles/d of NaCl (CON) or 10 moles/d of sodium acetate (ACE) (n = 8 per treatment). Least square means that do not share a superscript are significantly different (*p* < 0.05).

Plasma NEFA concentration was greater in ACE treatments (*p* = 0.02) and greater before feeding (AM; *p* < 0.001, Table 4). A tendency for an interaction between ruminal infusion and time was identified (*p* = 0.13), with greater plasma NEFA in the AM versus PM for ACE compared to CON (*p* = 0.05; Figure 3).

Treatment interactions were detected for plasma BHB (Table 4). Ruminal acetate infusions increased plasma BHB by 67% in the HF diet (*p* = 0.05) but did not affect BHB in the LF diet (Figure 3).

Rumen pH was higher with the LF diet (*p* = 0.01) and ACE (*p* < 0.001), and in the AM (*p* < 0.001: Table 4). Overall, the HF diet had a lower rumen pH than the LF diet (6.47 ± 0.06 and 6.64 ± 0.06, respectively). Rumen pH was higher for the ACE (6.7 ± 0.06) versus CON (6.41 ± 0.06).

## 4. Discussion

### 4.1. Dry Matter Intake, Milk Yield, and Milk Composition

The main objective of this study was to investigate the interaction between acetate supply and diet fermentability using equal forage-to-concentrate ratio diets, in European Holstein Friesian dairy cows. The absence of an interaction effect between diet fermentability and acetate infusion on milk fat yield, and therefore the rejection of our hypothesis, may be attributed to reductions in DMI and milk yield. Consequently, we explore these factors alongside the main effects of dietary and infusion treatments, aiming to identify potential limitations and suggest future research directions.

Variable effects of acetate supply on DMI and milk yield have been reported when providing exogenous acetate at a dose of 10 moles of sodium acetate/d (ruminally infused or mixed in a TMR). The dose of acetate infused was based on the titration study of Urrutia and Harvatine [8], where milk fat yield and the net transfer of ruminally infused acetate to milk fat were maximal at 10 moles of acetate/d. However, in the current study, ACE reduced intake and milk yield, as compared to CON, in a similar pattern (Figure 2). Urrutia et al. [4] and Matamoros et al. [13] observed a 9 and 6% increase in DMI, respectively. Matamoros et al. [13] did not observe any interaction between dietary fiber level and acetate on DMI or milk yield, when feeding 10 moles of sodium acetate/d in TMR diets that contained contrasting levels of dietary NDF (29 or 33%) and starch (28 or 21%) achieved by altering the forage-to-concentrate ratio (55:45 or 70:30). Maxin et al. [31] reported an 11% reduction in DMI and a 7% reduction in milk yield during 13 d of ruminal infusion of 18 moles acetate/d. Other studies infusing acetate into the rumen or mixing it with the diet have shown no effect of acetate on DMI and milk yield in lactating Holstein cows averaging above 30 kg of milk/d [8,13,21,30,32].

Regulation of feed intake is complex and involves integration of short- and long-term mechanisms, with the liver playing an important role in nutrient sensing to regulate meal size and frequency [33]. In that sense, propionate, but not acetate, is understood to reduce feed intake as proposed by the hepatic oxidation theory, as propionate is an anaplerotic metabolite [34]. However, continuous infusion of sodium acetate in the rumen allows constant acetate availability for use by extrahepatic tissues; therefore, propionate and other fuels are likely spared, which may explain the reduction in DMI observed in this study. The nutrient and energy requirements of the cows used in the current study were lower than those used previously, and ACE also reduced milk yield and thus energy requirements [8]; therefore, we propose that DMI was reduced by acetate through short-term mechanisms induced through sparing propionate or other fuels. Another possible mechanism to explain how acetate decreased DMI is through butyrate, as butyrate is an insulin secretagogue and insulin induces satiety and modifies hepatic oxidation of fuels. Acetate treatments increase plasma BHB [10,35], consistent with the response observed in the current study. Therefore, increased insulin release from pancreas would be expected; however, we did not measure circulating insulin, limiting the interpretation of this potential mechanism.

The integration of dietary energy intake and energy provided by acetate infusions, with energy lost in milk synthesis, allows for the calculation of apparent net energy balance. In our data, this balance was reduced in ACE treatments, reflecting the overall greater magnitude of DMI reduction in comparison to the decrease in milk yield in the ACE treatment. Further research should address expected responses to acetate supply dosage at different cow production levels and investigate factors affecting the responses of nutrient balances.

Milk fat concentration and yield have consistently increased with acetate supplementation across earlier studies (reviewed by [6,36]), and at the 10 moles/d dose, responses have ranged from 0.12 to 0.43 percentage units and from 90 to 220 g/d, for milk fat concentration and yield, respectively [4,5,8,13], with the lower response in yield observed when sodium acetate was fed in a TMR compared to a sodium bicarbonate control [4] and the high-end response corresponding to short-term (4 d) ruminal infusions of sodium acetate [8].

We hypothesized that acetate infusion would increase milk fat yield in highly fermentable diets, but instead, it reduced milk yield. However, milk fat concentration showed a numerically greater response to acetate than in previous studies (0.54 percentage units in HF), with a tendency to increase in the HF diet. Despite different experimental approaches to achieve contrasting dietary fiber levels, contrary to our hypothesis, our results align with those of Matamoros et al. [13], who also found no interaction between acetate and dietary fiber in milk fat yield. A key distinction between both studies lies in milk trans-10 C18:1, a well-established biomarker of biohydrogenation-induced milk fat depression [37]. In our trial, trans-10 C18:1 remained consistently low (0.14–0.15% of milk FA; Table 3), suggesting a more stable ruminal environment and reduced risk for diet-induced milk fat depression. In contrast, Matamoros et al. [13] observed higher concentrations (0.4–0.6%), even in high-fiber diets. This disparity suggests that, in their study, the elevated trans-10 C18:1 may have suppressed de novo FA synthesis, potentially masking any incremental effects of acetate supplementation. At this point, we are unsure of the reasons explaining the difference between the two studies in trans-10 C18:1 content. This interpretation should be supported by future research, which should provide a better understanding of how dietary composition and the ruminal environment interact to modulate the response to acetate infusion.

We reported a reduction in milk protein synthesis. Similarly, Matamoros et al. [13] reported a 3.3% reduction in milk protein concentration in low- and high-fiber diets supplemented with sodium acetate in a TMR, and Sheperd and Combs [30] reported a 5% reduction in milk protein concentration during long-term infusion of acetate in cows fed high-fiber diets. In those studies, the reductions in milk protein concentration were accompanied by greater DMI and milk yield, suggesting a dilution effect due to increased milk yield. Maxin et al. [31] also reported a 9.1% reduction in milk protein yield during short-term sodium acetate infusion, accompanied by reduced DMI and milk yield, similar to the responses observed in our study. The reduction in milk protein synthesis in our study may be explained by reduced nutrient intake or by a shift in nutrient partitioning within the mammary gland, or by both. Upregulation of milk fat synthesis, as evidenced by the increase in milk fat concentration, may have diverted nutrients and energy away from milk protein synthesis, prioritizing synthesis of FA over protein. This hypothesis aligns with the concept of nutrient competition within the mammary gland, where increased availability of a specific precursor (e.g., acetate) can alter the balance of milk component synthesis.

### 4.2. Milk Fatty Acid Profile and Rumen pH

Even though the milk fat yield remained unchanged across treatments, the profile of FA contained in milk fat was affected by the treatments. Our results support that acetate is a limiting factor for the elongation of de novo-synthetized FA in the mammary gland to C16:0, as evidenced by the greater C16:0 content in ACE, at both levels of diet fermentability and fiber. This suggests that under conditions of adequate fiber, acetate availability becomes a key driver of milk fat synthesis, potentially redirecting nutrients and energy away from other processes such as milk protein synthesis.

Consistent with the current report, acetate supply has consistently increased the milk content of mixed-origin FA, mainly through palmitic acid in various studies [4,5,8,13]. Acetate plays a fundamental role in milk FA synthesis in the mammary gland, and its supply indeed promotes the elongation of de novo-synthetized FA to palmitic acid, through yet poorly understood mechanisms [38]. An interesting observation that to our knowledge has not been reported before in animal trials is the increase in yield and content of trans-11 C18:1 and the increased content of stearic acid (C18:0) in ACE, as well as in the LF diet. We attribute the increase in content of preformed FA in the LF diet to the increased milk concentration of C18:0, trans-11 C18:1, and cis-9 C18:1 (Table 3). Vaccenic acid (trans-11 C18:1) is the most abundant trans C18:1 FA in milk [39], and it is the FA intermediate of the last step of biohydrogenation of unsaturated 18-carbon FA to stearic acid. The transfer and content of dietary preformed FA, such as vaccenic and stearic acid, to milk are influenced by diet composition, rumen passage rate, duodenal absorption, mammary gland uptake, and desaturase activity [40]. Greater concentrate inclusion and reduced pH resulted in reduced outflow of vaccenic acid in a continuous culture experiment [11]. In the same experiment, a greater outflow of C18:0 was observed at higher pH (6.4 vs. 5.6). It is likely that the increased content of trans-11 C18:1 we observed in ACE and in the LF diet relates to rumen pH, which was greater in ACE and also in the LF diet. The greater pH in the LF diet is explained by the higher starch and lower NDF content in the HF diet as compared to the LF diet (Table 1), which allows more substrate for VFA production and consequently reduced rumen pH [41]. The greater rumen pH in the ACE treatment was likely due to the buffering capacity of sodium acetate, which has been reported before [8]. Similar to our results, Alzahal et al. [42] reported increased greater mean rumen pH along with greater milk C18:0 content (2.5 percentage units) in a high-fiber diet (40% NDF, 13% starch, on DM basis), as compared to a low-fiber diet (33% NDF, 24% starch, on a DM basis). Rumen pH has been shown to alter the profile of 18-carbon FA through inhibition of fibrolytic bacteria that participate in biohydrogenation processes [16]. The increase in trans-11 C18:1 content in milk is potentially beneficial for consumers, as it can be converted into conjugated linoleic acid, which has demonstrated anticarcinogenic and other health benefits [43]. Because responses in milk vaccenic acid to acetate supply have not been previously reported, more research is needed to confirm this response under a greater variety of conditions.

### 4.3. Plasma Metabolites

The variation in plasma glucose relative to feeding time in cows fed once daily was expected, with the reduction in glycemia post feeding likely due to increased insulin secretion following cephalic stimulation of intake and high rates of propionate absorption during the active fermentation period [44].

Plasma NEFA originating from lipolysis in dairy cows is expected before feeding [45], with high energy demand or greater negative energy balance being associated with greater NEFA before feeding [46]. In contrast to our result, several studies have shown no effects of acetate infusion or acetate feeding on plasma NEFA [4,8,21]. However, in non-lactating cows, short-term infusion of 7 mol/d of sodium acetate increased plasma NEFA by 12%, while no changes in DMI were observed [47]. Also, Matamoros et al. [5] observed a tendency for increased NEFA when feeding sodium acetate and sodium bicarbonate in a TMR, as compared to a non-supplemented control TMR. The increased NEFA observed in the current study is likely a lipolysis response to reduced DMI and decreased apparent energy balance.

Increased plasma BHB is expected when acetate is being supplied in a TMR [5] or ruminally infused [8,21], through synthesis from acetate in the rumen wall [10,35]. Diets that contain more starch are expected to result in greater production of butyrate and propionate, compared to acetate in the rumen [48], and the HF diet contained 5% more starch than the LF diet. Although we did not measure rumen SCFA concentration or production, we attribute the interaction observed in plasma BHB to the combined effects of acetate supply on rumen wall BHB synthesis and availability of dietary starch on ruminal butyrate synthesis.

Also, a greater increase in plasma BHB was detected in the PM (6 h post feeding) when compared to AM, for the HF diet, than for the LF diet (interaction *p* = 0.08; Figure 3), similar to Matamoros et al. [13]. It is likely that the greater difference in plasma BHB observed between AM and PM in the HF diet, as compared to LF, resulted from increased ruminal butyrate production from the diet and further absorption and conversion to BHB in the rumen wall. It is well known that butyrate provides half the initial 4-carbons for milk FA synthesis [49,50]; therefore, butyrate is an important precursor for milk fat, as also shown by [51], who reported a 90 g increase in milk fat yield when supplementing dietary butyrate (1.1% diet DM). The interaction between diet fermentability and ACE observed in the current study for plasma BHB matches the tendency for the interaction observed between diet fermentability and ACE for milk fat concentration and supports the important role of butyrate on milk fat synthesis. Another observation from our data is that plasma NEFA was lower, and therefore lipolysis was likely also lower, when plasma BHB was greater, in accordance with the lipolytic suppression effect of BHB through activation of the metabolic sensor GPR109A [52].

## 5. Conclusions

The main objective of this study was to investigate the interaction between diet fermentability and acetate supply on milk fat synthesis. Ruminal infusions of acetate depressed intake to an extent that reduced milk yield; therefore, the main effect of acetate and its interaction with diet fermentability was confounded by reduced nutrient intake. Exogenous acetate supply, along with its interaction with diet fermentability level, failed to increase milk fat yield; however, acetate supply increased milk fat concentration, through increased synthesis of palmitic acid, and tended to increase milk fat content in the more fermentable diet. Interestingly, acetate favored milk fat synthesis over milk protein synthesis. Reduced diet fermentability increased the content of preformed FA in milk, while increasing rumen pH. This study supports the role of exogenous acetate on the regulation of milk fat synthesis.

## Figures and Tables

**Table 1:** Composition of experimental diets ^1,2^.

Item	HF	LF
Ingredients, g/100 g DM		
Corn silage	65	65
Ground corn	17.4	5.4
Soybean meal	15.5	16.5
Oat hulls	-	11
Mineral–vitamin mix ^3^	1.25	1.25
NPN ^4^	0.85	0.85
Nutrients, g/100 g DM		
NDF	34.6 ± 1.1	38.6 ± 1.1
ADF	21.6 ± 1.1	23.8 ± 0.8
CP	16.1 ± 0.2	16.8 ± 0.4
Starch	21.2 ± 1.1	16.3 ± 1.3
Ether extract	2.14 ± 0.21	1.98 ± 0.2
Ash	6.26 ± 0.24	6.60 ± 0.24
NFC	40.9 ± 1	36.0 ± 1.1
Digestibility ^5^	77.9 ± 1.9	73.8 ± 1.3
NEL ^6^, Mcal/kg	1.58 ± 1.9	1.51 ± 0.02

^1^ Forage-to-concentrate ratio, 65:35. ^2^ Diet treatments were a high-fermentability diet (HF) or low-fermentability diet (LF). ^3^ Composition (DM basis): 20% Ca; 5% P; 5% Mg; 6% Na; 9% Cl; 2% S; 10 mg/kg of Co; 1200 mg/kg of Cu; 100 mg/kg I; 2000 mg/kg Mn; 35 mg/kg of Se; 5500 mg/kg of Zn; 100,000 IU/kg of vitamin A; 50,000 IU/kg of vitamin D; and 1000 IU/kg of vitamin E (Vetersal Alta Producción, Veterquimica, Santiago, Chile). ^4^ Non-protein nitrogen fed as a slow-release urea (Optigen, Alltech Inc., Lexington, KY, USA; 259% CP, DM basis). ^5^ Diet digestibility was estimated as follows: 100 × [(Initial OM-Residual OM)÷Initial DM]. ^6^ Diet NE_L_ calculated as in [20].

**Table 2:** Effect of diet fermentability and acetate supply on DMI, milk yield, and milk components.

Variable	Treatments ^1^				SE	*p*-Value ^2^				
	HF		LF							
	CON	ACE	CON	ACE		F	I	T	F × I	I × T
DMI, kg/d	21.6	19.4	21.7	18.6	1.1	0.74	0.01	0.001	0.67	0.003
Milk yield, kg/d	20.6	18.7	20.9	18.5	1.0	0.93	<0.001	0.015	0.40	0.03
Milk fat %	4.38	4.91	4.37	4.63	0.12	0.08	<0.001	0.29	0.12	0.58
Milk fat yield, kg/d	0.90	0.91	0.92	0.89	0.03	0.99	0.66	0.40	0.29	0.71
Milk protein %	3.76	3.62	3.74	3.65	0.04	0.99	0.001	0.87	0.27	<0.001
Milk protein, kg/d	0.78	0.68	0.78	0.68	0.03	0.85	0.001	0.02	0.78	0.03
Apparent NEL Balance ^3^, Mcal/d	32.7	29.3	31.1	27.0	2.59	0.21	0.03	0.003	0.81	0.12

^1^ Treatments were arranged in a 2 × 2 factorial design of diet fermentability [high fermentability (HF) or low fermentability (LF)] and ruminal infusions of 10 moles/d of NaCl (CON) or sodium acetate (ACE). ^2^ Diet fermentability (F), ruminal infusion (I), day of treatment (T), and their interactions. ^3^ Apparent NE_L_ balance was calculated as follows: (Dietary intake NE_L_ + Infusion NE_L_)—Milk NE_L_, with milk NE_L_ = Milk yield (kg/d) × [((0.0929 × fat %) + (0.0547 × protein %) + 0.192] as in NRC (2001) [20], and infusion NE_L_ was calculated as 95% of ME based on the heat of combustion of acetate (0.209 Mcal/mol), as in Sheperd and Combs [30].

**Table 3:** Effect of diet fermentability and acetate supply on milk fatty acids (FAs).

Milk FA	Treatments ^1^				SE	*p*-Value ^2^		
	HF		LF					
	CON	ACE	CON	ACE		F	I	F × I
FA content by source ^3^, %								
De novo	31.6	30.0	31.7	29.4	1.1	0.68	0.01	0.60
Mixed	37.0	40.9	37.5	38.9	1.2	0.55	0.03	0.31
Preformed	22.8	23.7	24.9	26.4	1.1	0.04	0.28	0.77
OBCFA ^4^	3.50	3.08	3.56	3.40	0.15	0.19	0.05	0.36
FA yield by source, g/d								
De novo	258	251	254	231	20	0.50	0.36	0.64
Mixed	301	341	306	307	28	0.57	0.42	0.44
Preformed	211	213	220	216	16	0.52	0.95	0.75
OBCFA	28.9	25.4	29.4	26.2	2.3	0.76	0.13	0.94
Specific FA of interest, % of FA								
C16:0	35.5	39.4	36.0	37.5	1.1	0.54	0.02	0.26
C18:0	7.21	7.96	7.94	8.82	0.37	0.02	0.02	0.83
C18:1 trans-10	0.15	0.14	0.15	0.15	0.01	0.23	0.49	0.28
C18:1 trans-11	0.37	0.46	0.44	0.54	0.03	0.002	<0.001	0.80
Total trans C18:1	0.99	1.10	1.10	1.20	0.07	0.04	0.03	0.98

^1^ Treatments were arranged in a 2 × 2 factorial design of diet fermentability [high fermentability (HF) or low fermentability (LF)] and ruminal infusions of 10 moles/d of NaCl (CON) or sodium acetate (ACE). ^2^ Diet fermentability (F), ruminal infusion (I), and their interactions. ^3^ Fatty acids by source were calculated by adding all straight-chain and even-carbon FA < 16 carbons (de novo); 16-carbon FA (mixed); and >16C (preformed). Yields were calculated using milk fat yield and milk FA composition corrected for glycerol content. ^4^ Odd- and branched-chain FA.

**Table 4:** Effect of diet fermentability and acetate supply on plasma concentration of metabolites and rumen pH.

Variable	Treatments ^1^					*p*-Value ^2,3^					
	HF		LF		SE						
	CON	ACE	CON	ACE		F	I	T	F × I ^4^	F × T	I × T
Glucose, mg/dL	52.9	52.2	51.7	52.0	1.3	0.53	0.83	0.001	0.63	0.74	0.41
AM	55.5	53.4	53.4	53.5	1.7						
PM	50.2	50.9	50.1	50.5	1.5						
NEFA, μEq/L	82.5	101.3	81.3	123.9	12	0.37	0.02	<0.001	0.32	0.29	0.13
AM	98.9	119.5	94.2	169.0	21						
PM	66.1	83.2	68.5	78.8	9.5						
BHB ^4^, μM	0.65 ^b^	1.08 ^a^	0.62 ^b^	0.77 ^b^	0.1	0.05	0.002	<0.001	0.08	0.08	0.12
AM	0.42	0.70	0.47	0.56	0.09						
PM	0.87	1.46	0.77	0.98	0.14						
Rumen pH	6.31	6.63	6.51	6.77	0.07	0.01	<0.001	<0.001	0.65	0.66	0.26
AM	6.77	6.9	6.83	7.1	0.12						
PM	5.86	6.36	6.19	6.44	0.08						

^1^ Treatments were arranged in a 2 × 2 factorial design of diet fermentability [high fermentability (HF) or low fermentability (LF)] and ruminal infusions of 10 moles/d of NaCl (CON) or sodium acetate (ACE). ^2^ Diet fermentability (F), ruminal infusion (I), time of day (T), and their interactions. ^3^ There were no significant 3-way interactions or tendencies towards them (F × I × T). ^4^ When an interaction is significant (*p* < 0.1), LSMs that do not share a superscript are significantly different (*p* < 0.05).

## Data Availability

The original contributions presented in this study are included in the article. Further inquiries can be directed to the corresponding author.

## Supplementary Materials

The following supporting information can be downloaded at https://www.mdpi.com/article/10.3390/ani15131931/s1, Table S1: Effect of diet fermentability and acetate supply on yield of milk fatty acids (FA); Table S2: Effect of diet fermentability and acetate supply on content of milk fatty acids (FA).

## Abbreviations

The following abbreviations are used in this manuscript:

HF	High fermentability
LF	Low fermentability
ACE	Acetate treatment
CON	Control treatment
DMI	Dry matter intake
SCFA	Short-chain fatty acid
TMR	Total mixed ration
NDF	Neutral detergent fiber
DM	Dry matter
CP	Crude protein
ADF	Acid detergent fiber
OM	Organic matter
NFC	Non-fiber carbohydrates
FA	Fatty acid
AM	Before midday
PM	After midday
NEFA	Non-esterified fatty acid
BHB	β-hydroxybutyrate
