# Supplementary material for: Interaction Between Ruminal Acetate Infusion and Diet Fermentability on Milk Fat Production in Dairy Cows
Source: Animals (Basel). 2025 Jun 30;15(13):1931. doi: 10.3390/ani15131931 (PMC12248817; doi:10.3390/ani15131931)
Supplement: Supplementary file 1 [file animals-15-01931-s001.zip › animals-3714935-supplementary.pdf]

Supplementary Table S1. Effect of diet fermentability and acetate supply on yield of milk fatty acids (FA)

Milk FA, g/d	Treatments[1]				SE	*p*-Value[2]		
	HF		LF					
	CON	ACE	CON	ACE		F	I	F × I
C4:0	38.4	40.7	39.9	38.4	4.1	0.90	0.91	0.58
C6:0	21.4	21.3	21.6	19.7	2.0	0.65	0.54	0.60
C8:0	12.0	11.3	11.6	10.3	1.0	0.40	0.22	0.68
C10:0	28.9	26.5	27.0	23.7	2.4	0.20	0.11	0.81
C12:0	35.7	32.1	32.9	28.2	2.8	0.13	0.06	0.79
C14:0	104	103	104	96.4	7.8	0.58	0.54	0.65
C14:1, cis-9	14.3	12.8	14.3	11.9	1.0	0.65	0.04	0.63
C16:0	289	329	294	296	26	0.56	0.39	0.43
C16:1, cis-9	12.1	12.2	12.1	11.1	1.5	0.69	0.71	0.66
C18:0	58.4	65.1	63.7	67.9	5.1	0.19	0.08	0.66
C18:1 t4	0.03	0.05	0.05	0.03	0.01	0.97	0.68	0.03
C18:1 t5	0.05	0.05	0.07	0.07	0.02	0.19	0.98	0.90
C18:1 t6-8	1.15	1.15	1.27	1.24	0.12	0.15	0.83	0.83
C18:1 t9	0.83	0.84	0.87	0.86	0.08	0.58	0.95	0.85
C18:1 t10	1.25	1.18	1.26	1.22	0.14	0.80	0.51	0.87
C18:1 t11	3.11	4.08	3.52	4.04	0.33	0.31	0.001	0.19
C18:1 t12	1.71	1.70	1.79	1.72	0.16	0.59	0.68	0.76
C18:1 c9	99.6	103	110	107	8.1	0.21	0.99	0.54
C18:1 c11	3.22	3.25	3.06	3.12	0.26	0.53	0.81	0.94
C18:1 c12	0.92	0.78	0.91	0.80	0.08	0.96	0.03	0.78
C18:2 c9c12	9.29	8.95	8.17	7.58	0.66	0.03	0.35	0.80
C18:2 c9t11	0.71	0.66	0.78	0.60	0.08	0.89	0.07	0.29
C18:3 n6	0.92	1.00	1.02	1.04	0.08	0.22	0.39	0.53
C18:3 n3	0.80	0.83	0.80	0.80	0.08	0.85	0.84	0.86

[1]Treatments were arranged in a 2x2 factorial design of diet fermentability [high fermentability (HF) or low fermentability (LF)] and ruminal infusions of 10 moles/d of NaCl (CON) or sodium acetate (ACE). [2]Diet fermentability (F), ruminal infusion (I), and their interactions.

Supplementary Table S2. Effect of diet fermentability and acetate supply on content of milk fatty acids (FA)

Milk FA, g/100g	Treatments[1]				SE	*p*-Value[2]		
	HF		LF					
	CON	ACE	CON	ACE		F	I	F × I
C4:0	4.73	4.88	4.93	4.85	0.23	0.65	0.86	0.51
C6:0	2.62	2.56	2.67	2.49	0.10	0.89	0.14	0.48
C8:0	1.46	1.35	1.45	1.31	0.06	0.48	0.004	0.69
C10:0	3.53	3.17	3.39	3.02	0.19	0.07	<.0001	0.90
C12:0	4.42	3.98	4.13	3.58	0.24	0.002	<.0001	0.48
C14:0	12.8	12.4	12.9	12.3	0.42	0.96	0.04	0.65
C14:1, cis-9	1.77	1.51	1.82	1.57	0.10	0.38	0.000	0.98
C16:0	35.5	39.4	36.0	37.5	1.1	0.54	0.02	0.26
C16:1, cis-9	1.51	1.48	1.51	1.43	0.13	0.79	0.53	0.78
C18:0	7.21	7.96	7.94	8.82	0.37	0.02	0.02	0.83
C18:1 t4	0.004	0.006	0.006	0.004	0.002	0.74	0.96	0.03
C18:1 t5	0.006	0.006	0.008	0.009	0.002	0.08	0.85	0.98
C18:1 t6-8	0.140	0.142	0.156	0.161	0.01	0.000	0.39	0.73
C18:1 t9	0.102	0.103	0.107	0.112	0.01	0.13	0.50	0.64
C18:1 t10	0.153	0.143	0.153	0.155	0.01	0.23	0.49	0.28
C18:1 t11	0.372	0.461	0.445	0.543	0.03	0.002	0.000	0.80
C18:1 t12	0.210	0.209	0.221	0.221	0.01	0.11	0.92	0.96
C18:1 c9	12.2	12.5	13.6	14.1	0.80	0.047	0.53	0.86
C18:1 c11	0.39	0.40	0.38	0.41	0.02	1.00	0.45	0.58
C18:1 c12	0.11	0.10	0.11	0.10	0.01	0.55	0.03	0.33
C18:2 c9c12	1.14	1.09	1.01	1.00	0.06	0.06	0.58	0.71
C18:2 c9t11	0.09	0.08	0.10	0.09	0.00	0.01	0.03	0.95
C18:3 n6	0.11	0.12	0.13	0.13	0.01	0.003	0.07	0.99
C18:3 n3	0.10	0.10	0.10	0.11	0.01	0.77	0.43	0.77

[1]Treatments were arranged in a 2x2 factorial design of diet fermentability [high fermentability (HF) or low fermentability (LF)] and ruminal infusions of 10 moles/d of NaCl (CON) or sodium acetate (ACE). [2]Diet fermentability (F), ruminal infusion (I), and their interactions.
